# Evaluation of Spray-Dried Bovine Hemoglobin Powder as a Dietary Animal Protein Source in Nile Tilapia, *Oreochromis niloticus*

**DOI:** 10.3390/ani12223206

**Published:** 2022-11-18

**Authors:** Shimaa A. Amer, Mahmoud Farahat, Tarek Khamis, Samar A. Abdo, Elsayed M. Younis, Abdel-Wahab A. Abdel-Warith, Rehab Reda, Sozan A. Ali, Simon J. Davies, Rowida E. Ibrahim

**Affiliations:** 1 Department of Nutrition & Clinical Nutrition, Faculty of Veterinary Medicine, Zagazig University, Zagazig 44511, Egypt; 2 Department of Pharmacology, Faculty of Veterinary Medicine, Zagazig University, Zagazig 44511, Egypt; 3 Biochemistry Department, Faculty of Veterinary Medicine, Zagazig University, Zagazig 44511, Egypt; 4 Department of Zoology, College of Science, King Saudi University, Riyadh 11451, Saudi Arabia; 5 Animal Wealth Development Department, Faculty of Veterinary Medicine, Zagazig University, Zagazig 44511, Egypt; 6 Department of Histology and Cytology, Faculty of Veterinary Medicine, Zagazig University, Zagazig 44511, Egypt; 7 School of Science and Engineering, National University of Ireland Galway, H91 TK33 Galway, Ireland; 8 Department of Aquatic Animal Medicine, Faculty of Veterinary Medicine, Zagazig University, Zagazig 44511, Egypt

**Keywords:** Nile tilapia, spray-dried bovine hemoglobin, growth, protein absorption, immune-related genes

## Abstract

**Simple Summary:**

Animal by-product meals are considered the safest and most unexploited source of animal protein in the fish industry. This study evaluated the potential effects of dietary spray-dried bovine hemoglobin powder as an animal protein source on growth, digestive enzyme activity, intestinal histomorphology, immune status, and economic efficiency in Nile tilapia, *Oreochromis niloticus.* The study demonstrated that spray-dried bovine hemoglobin powder could be used as a protein source for up to 10% of the diets of Nile tilapia (*Oreochromis niloticus*) for better growth and immune status of the fish.

**Abstract:**

The present study evaluated the potential effects of dietary inclusion of spray-dried bovine hemoglobin powder (SDBH) on the growth, gene expression of peptide and amino acid transporters, insulin growth factor-1 (*IGF-1*) and *myostatin*, digestive enzymes activity, intestinal histomorphology and immune status, immune-related gene expression, and economic efficiency in Nile tilapia, *Oreochromis niloticus*. Two hundred twenty-five fingerlings (32.38 ± 0.05 g/fish) were distributed into five treatments with five dietary inclusion levels of SDBH: 0, 2.5, 5, 7.5, and 10% for a ten-week feeding period. Dietary inclusion of SDBH linearly increased the final body weight (FBW), total weight gain (TWG), specific growth rate (SGR), and protein efficiency ratio (PER). Additionally, a linear decrease in feed conversion ratio (FCR) and daily feed intake relative to the daily BW was reported in the highest inclusion levels (7.5 and 10%). Dietary inclusion of SDBH was associated with a significant increase in the intestinal villous height (VH), villous width (VW), villous height: crypt depth ratio (VH: CD), and muscle coat thickness (MCT), with the highest values reported in SDBH7.5 group. Increased serum growth hormone levels and decreased serum leptin hormone levels were also reported by increasing the SDBH level. The serum glucose level was decreased in the SDBH7.5 and SDBH10 groups. The digestive enzymes’ activity (amylase and protease) was increased by increasing the SDBH inclusion level. An up-regulation in the expression of peptide and amino acid transporters, *IGF-1,* and down-regulation of *myostatin* was reported in the SDBH2.5 to SDBH7.5 groups. Spleen sections showed more lymphoid elements, especially in the SDBH2.5 and SDBH7.5 groups. The SDBH inclusion increased the serum lysozyme activity, nitric oxide (NO), and complement 3 (C3) levels, with the highest values recorded in the SDBH5 group. The phagocytic % and the phagocytic index were increased by increasing the SDBH inclusion %. The expressions of immune-related genes (transforming growth factor-beta (*TGF-β*), Toll-like receptor 2 (*TLR2*), and interleukin *10* (*IL10*)) were up-regulated by SDBH inclusion with the highest expression in the SDBH5 group. Economically, the feed costs and feed costs/kg gain were linearly decreased in the SDBH7.5 and SDBH10 diets. In conclusion, spray-dried bovine hemoglobin powder could be used as a protein source for up to 10% of the diets of Nile tilapia for better growth and immune status of fish.

## 1. Introduction

Aquaculture production has expanded quickly throughout the past decade. Nile tilapia is the most important species of freshwater fish in tropical and subtropical regions [1]. It is an omnivorous fish that can tolerate various environmental conditions. It is an important economic species, because it accepts both natural and synthetic feeds [2]. Aquatic culture is growing worldwide, raising the need for feed components to encourage production [3]. Half of the aquaculture production costs have been reported to be feed costs [4]. Since protein is the most expensive feed component [5], the nutritional value of fish feed depends mainly on the protein quality of the ingredients used in feed formulation [6]. The market price of fish meal (FM) has risen recently due to the increased need for FM in aquaculture feed manufacturing and the depletion of marine fishery resources [7]. Therefore, assessing low-FM feed preparations for the target cultured species is essential.

The use of dietary plant protein supplements is usually restricted because of essential amino acid deficiency and the presence of anti-nutrient factors [8]. Animal protein components are characterized by a high content of digestible protein with a good amino acid profile, digestible dry matter, and digestible energy [9]. Earthly animal by-product meals such as blood meals and blood products are considered the safest and essentially most unexploited available source of animal protein in the aquatic feed industry. 

Spray-dried blood and spray-dried plasma proteins are feed ingredients of high value for farm animals [10]. Bovine hemoglobin powder (BH) is produced by splitting hemoglobin from hygienically gathered and isolated eligible healthy bovine blood. Spray drying (low-temperature processing method) resulted in slight biological degradation of amino acids in the high protein-containing BH (92% of dry matter) [11] and high lysine and leucine contents. However, it still shows a low isoleucine content [12]. Dried bovine hemoglobin powder improved fish growth, health, antioxidant capacity, blood hematology, and increased resistance to *Aeromonas veronii* [13]. Due to the aforementioned characteristics, SDBH could be a potential protein ingredient in fish diets. Therefore, this study evaluated the SDBH as a possible protein ingredient in Nile tilapia diets and assessed the effects of this inclusion on the growth, growth-related genes, digestive enzyme activity, blood biochemical parameters, intestinal histomorphology, immune status, and immune-related genes in Nile tilapia, *O. niloticus.*

## 2. Materials and Methods

### 2.1. Fish and Experimental Design

The current experiment was conducted in the Aquatic Animal Medicine department laboratory, Faculty of Veterinary medicine, Zagazig University, Egypt. The experimental procedures were approved by the Institutional Animal Care and Use Committee (ZU-IACUC) of Zagazig University, Egypt (Approval No. ZU-IACUC/2/F/136/2022). 

Two hundred twenty-five fingerlings (32.38 ± 0.05 g/fish) were bought from Abbassa Fish Hatchery, Sharkia Province, Egypt, and used in the current experiment. Two weeks before the experiment, the fish were acclimatized to laboratory conditions by feeding on the basal diet in the first week and then on the experimental diets in the second week. 

Following acclimatization, fish were stocked in fifteen static glass aquariums (50 cm × 40 cm × 60 cm). The tank water was changed daily by 25% and completely changed twice weekly. Throughout the experimental period, the water quality was maintained within the recommended limits according to APHA [14], with no significant variations owing to SDBH inclusion: dissolved oxygen (6.5–7.02 mg L^−1^), temperature (26.5–26.9 °C), pH (6.3–6.7), and unionized ammonia (0.026–0.046 mg L^−1^). The fish were haphazardly distributed to five treatments in triplicate (15 fish/replicate) for a ten-week feeding period. The experimental treatments comprised isocaloric isonitrogenous basal diets with five inclusion levels of SDBH (ACTIPRO^®^ 95 BHS, Zwevezele, Belgium) (0, 2.5, 5, 7.5, or 10%), and SDBH0 was kept as the control diet. The groups will be SDBH0, SDBH2.5, SDBH5, SDBH7.5, and SDBH10. During the adaptation and experimental period, fish were fed manually twice daily (at 9 am and 2 pm) until satiation on 2 mm pelleted diets that were formulated following NRC [5] (Table 1) and were analyzed for determination of moisture, crude protein, ether extract, and ash content following AOAC 2000 [15]. Daily monitoring of fish was done for any signs of disease or mortality.

### 2.2. Growth Performance Parameters

Fish were weighed to record the initial fish body weights at the beginning of the experiment, then the final body weight (FBW) and total feed intake (TFI) were recorded at the end of the feeding period. Average daily FI relative to daily BW (g FI/g BW/day) was also calculated. The following growth indices were determined according to Castell and Tiews [16];

$$\mathrm{Total}\;\mathrm{weight}\;\mathrm{gain}:\;\mathrm{TWG}\;\left(g/\mathrm{fish}\right)=\left(\mathrm{WT}-\mathrm{WI}\right)$$

where WT = final body weight (g/fish) and WI = initial weight (g/fish).

$$\mathrm{Average}\;\mathrm{daily}\;\mathrm{weight}\;\mathrm{gain}\;\left(\mathrm{ADWG}\right)\;\left(g/\mathrm{fish}/\mathrm{day}\right)=\frac{\mathrm{TWG}\;\left(g\right)}{\mathrm{Experimental}\;\mathrm{days}\;}.$$

$$\mathrm{Feed}\;\mathrm{conversion}\;\mathrm{ratio}\;\left(\mathrm{FCR}\right)=\frac{\mathrm{TFI}\;\mathrm{per}\;\tan k\;\left(g\right)}{\mathrm{TWG}\;\mathrm{of}\;\tan k\;\left(g\right)}$$

$$\mathrm{Specific}\;\mathrm{growth}\;\mathrm{rate}\;\left(\mathrm{SGR}\right)\left(\%/\mathrm{day}\right)=100\times \frac{\ln\;\mathrm{WT}\;-\;\ln\;\mathrm{WI}}{\mathrm{time}\;\mathrm{in}\;\mathrm{days}\;}$$

where ln is the natural logarithm. 

The protein efficiency ratio (PER) was calculated following Stuart and Hung [17].

$$\;\mathrm{PER}\;=\frac{\mathrm{TWG}\;\left(g\right)}{\mathrm{protein}\;\mathrm{intake}\;\left(g\right)}$$

### 2.3. Sample Collection

Blood was sampled from the caudal vein of fish (12 fish/group) in two aliquots at the end of the feeding period. The first aliquot was collected with anticoagulant to determine the phagocytic activity. The second one was collected without anticoagulant and centrifugated at 1075× *g* for 20 min to separate serum to measure the biochemical parameters. Samples from the intestine and spleen were collected for histological examination. Intestine, spleen, and muscle samples for gene expression studies were collected as 50 mg of tissue on 1 mL Quiazol (Qiagen, Hilden, Germany) and stored at −80 °C for total RNA extraction.

### 2.4. Digestive Enzyme Activity

The intestine with pyloric caeca was collected to determine pancreatic enzyme activity (amylase and protease). All methods were performed on ice to inactivate enzymes during the samples’ preparation, following Esmaeili et al. [18].

### 2.5. Histology

At the termination of the experiment, samples from the intestine and spleen were taken (9 fish/group) and fixed in 10% neutral buffered formalin. The samples were embedded in paraffin, and paraffin blocks were followed with tissue sections at 5 μ thickness by microtome Leica^®^ (Wetzlar, Germany) and routinely stained with hematoxylin and eosin (H&E). Slides were examined as described by Suvarna et al. [19]. 

### 2.6. Serum Biochemical Indices

Fish growth hormone and leptin hormone ELISA kits (MyBioSource Co., San Diego, CA, USA, Cat. Nos. MBS044656 and MBS021271, respectively) were used to determine growth hormone levels (GH) and leptin hormone, following the manufacturer’s instructions. Glucose was estimated using spectrum-bioscience kits (Egyptian Company for Biotechnology, Cairo, Egypt).

### 2.7. Immunological Indices

According to Ellis [20], the serum lysozyme activity was assessed by spectrophotometry based on the lysis of freeze-dried particles of *Micrococcus lysodeikticus*. The serum nitric oxide level was measured using a colorimetric assay following Montgomery and Dymock [21]. MyBioSource Co. ELISA kits Cat. No. MBS005953 were used to measure serum complement 3 (C3) following the producer’s guidelines [22]. According to the formula of Siwicki et al. [23], the phagocytic activity (%) and phagocytic index were calculated:

(1) $$\mathrm{Phagocytic}\;\mathrm{activity}\%=\frac{\mathrm{No}.\mathrm{of}\;\mathrm{phagocytic}\;\mathrm{cell}\;\mathrm{phagocytizing}\;\mathrm{bacteria}}{\mathrm{Total}\;\mathrm{no}.\mathrm{of}\;\mathrm{phagocytic}\;\mathrm{cells}\;\mathrm{counted}}\times 100$$

(2) $$\mathrm{Phagocytic}\;\mathrm{index}\;=\frac{\mathrm{Total}\;\mathrm{no}.\mathrm{of}\;\mathrm{phagocytized}\;\mathrm{bacteria}}{\mathrm{No}.\mathrm{of}\;\mathrm{phagocytic}\;\mathrm{cells}\;\mathrm{phagocytizing}\;\mathrm{bacteria}}$$

### 2.8. Quantitative Real-Time RT-PCR Analysis (qRT-PCR) 

For the gene expression study, the total RNA was extracted from 50 mg (muscle (2–3 cm white skeletal muscle below the dorsal fin), spleen, and intestine) of three fish per replicate (*n* = 9) with 1 mL Quiazol (Qiagen, Hilden, Germany) according to the manufacturer’s instruction. The cDNA was synthesized from 1 µg with a high-capacity reverse transcriptase kit (Applied Biosystem, Foster City, CA, USA) in a final reaction volume of 20 µL (10 µL master mix & 10 µL RNA sample containing 1 µg RNA). cDNA was diluted at 1:10 and stored in aliquots at −20 °C for qPCR reaction. The qPCR was done by real-time thermal cycler Rotor-Gene Q 2 plex (Qiagen, Hilden, Germany) [24]. Real-time quantitative PCR (RT-qPCR) analyses of immune-related genes in the spleen (*transforming growth factor-beta* (*TGF-β*), *Toll-like receptor 2* (*TLR2*), and interleukin 10 (IL-10)), peptide and AA transporters in the intestine (*solute carrier family 15 member 2* (*SLC15A2*) and *solute carrier family 26 member 6* (*SLC26A6*)), and growth-related genes in the muscle (insulin-like growth factor-1 (*IGF-1*) and myostatin) were performed with specified primers (Sangon Biotech, Beijing, China) using beta-actin (β-actin) as a reference gene, as described by Schmittgen and Livak [25]. These are listed in Table 2.

### 2.9. Economic Efficiency

Feed cost (USD) = The cost (USD/Kg diet), including SDBH (0.7 USD/Kg SDBH) × TFI (kg)

(3) $$\mathrm{Feedcost}/\mathrm{kg}\;\mathrm{gain}\;=\frac{\;\mathrm{Total}\;\mathrm{feed}\;\cos\mathrm{ts}\;\left(\mathrm{USD}\right)}{\mathrm{Total}\;\mathrm{weight}\;\mathrm{gain}\;\left(\mathrm{kg}\right)}\;$$

### 2.10. Statistical Analysis

The data were tested for normality and homogeneity; then ANOVA test was applied based on polynomial orthogonal contrasts. Equations of linear and quadratic regression were designed using SPSS Version 17 for Windows (SPSS Inc., Chicago, IL, USA). A post hoc Tukey’s test was used to determine differences among means, the variation in the data was expressed as pooled SEM, and the significance level was set at *p* < 0.05. 

## 3. Results

### 3.1. Growth Performance

There was a linear rise in the FBW, ADWG, TWG, SGR, and PER by SDBH inclusion (*p* < 0.01). In addition, dietary inclusion of SDBH resulted in a linear improvement in the FCR (*p* < 0.01). A linear decrease in the average daily feed intake relative to the daily BW (g FI/g BW/day) was observed in the SDBH7.5 and SDBH10 groups compared to the SDBH0 group (*p* = 0.02) (Table 3). The FBW was increased by 3.46, 5.58, 6.74, and 11.91% in the SDBH2.5, SDBH5, SDBH7.5, and SDBH10 groups, respectively. The FCR was improved by 5.6, 3.38, 16.52, and 22.85% in the SDBH2.5, SDBH5, SDBH7.5, and SDBH10 groups, respectively. 

### 3.2. Metabolic Function Indices

An increase in the growth hormone level and a decrease in the leptin hormone level were reported by increasing the level of inclusion (linear and quadratic, *p* ≤ 0.01). The serum glucose level was decreased linearly and quadratically in the SDBH7.5 and SDBH10 groups (*p* < 0.05) (Table 4).

### 3.3. Digestive Enzymes Activity

**Figure 1:**
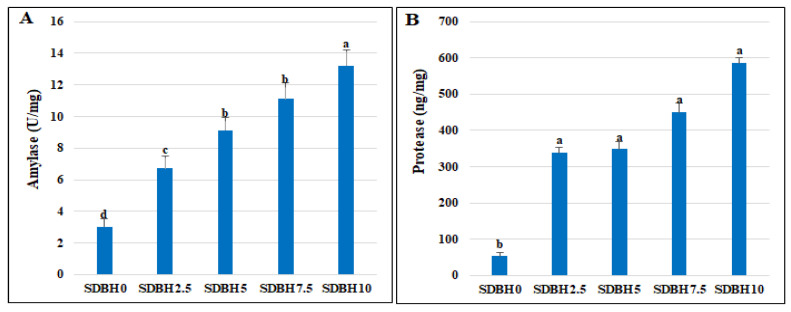
Impact of dietary SDBH inclusion on the digestive enzymes’ activity (amylase (**A**) and protease (**B**)) in *O. niloticus*. ^a,b,c,d^ The bars with different lowercase letters are significantly different (*p* < 0.05).

A linear increase in the amylase and protease activity was observed by increasing the SDBH inclusion level (*p* < 0.05) (Figure 1). The amylase activity increased by 122.18, 202.31, 267.54, and 338.07% in SDBH2.5, SDBH5, SDBH7.5, and SDBH10, respectively. The protease activity increased by 532.71, 551.40, 741.12, and 994.39% in SDBH2.5, SDBH5, SDBH7.5, and SDBH10, respectively.

### 3.4. Intestinal mRNA Expressions of Peptide and AA Transporters

**Figure 2:**
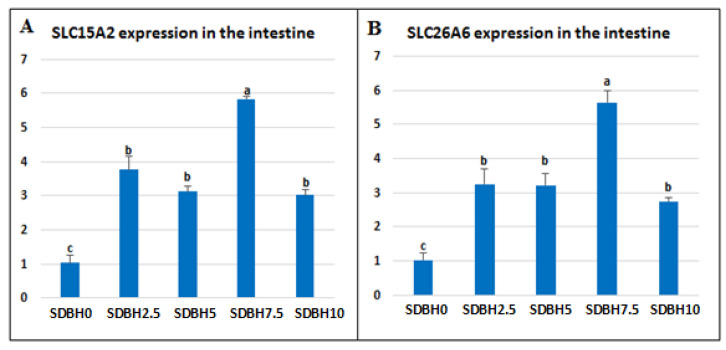
Impact of dietary SDBH inclusion on the intestinal expressions of peptide and AA transporters in *O. niloticus. SLC15A2*: solute carrier family 15 members 2 (**A**), *SLC26A6*: solute carrier family 26 members 6 (**B**). ^a,b,c^ The bars with different lowercase letters are significantly different (*p* < 0.05).

An up-regulation in the intestinal mRNA expression of *SLC15A2* and *SLC26A6* (*p* < 0.05) was reported with all SDBH inclusion levels compared to the control group (SDBH0), with the highest expression being in SDBH7.5 (Figure 2). Compared to the SDBH0 group, the intestinal mRNA expression of *SLC15A2* was boosted by 3.75-, 3.12-, 5.84-, and 3.01-fold in SDBH2.5, 5, 7.5, and 10, respectively. In addition, the intestinal mRNA expression of *SLC26A6* was boosted by 3.24-, 3.22-, 5.64-, and 2.73-fold in SDBH2.5, SDBH5, SDBH7.5, and SDBH10, respectively.

### 3.5. mRNA Expressions of IGF-1 and Myostatin in the Muscles

**Figure 3:**
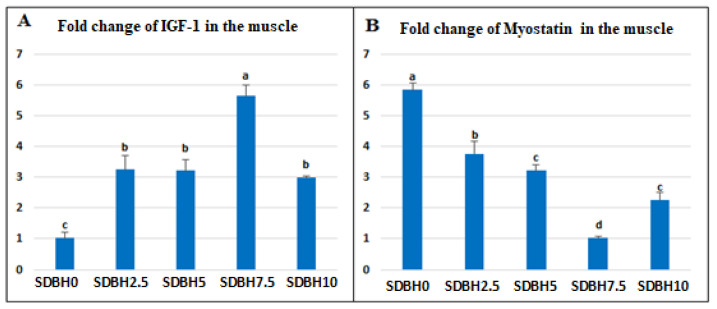
Impact of dietary SDBH inclusion on the expressions of insulin-like growth factor-1 (*IGF-1*) (**A**) and *Myostatin* (**B**) in the muscle of *O. niloticus.* ^a,b,c,d^ The bars with different lowercase letters are significantly different (*p* < 0.05).

An up-regulation in the muscle mRNA expression of *IGF-1* A and down-regulation in the muscle mRNA expression of *myostatin* (*p* < 0.05) were reported from SDBH2.5 to SDBH10; the highest *IGF-1* expression was reported in the SDBH7.5 group (Figure 3). Compared to the SDBH0 group, the muscle mRNA expression of *IGF-1* was boosted by 3.24-, 3.22-, 5.64-, and 2.98-fold in SDBH2.5, SDBH5, SDBH7.5, and SDBH10 groups, respectively. In addition, the muscle mRNA expression of *myostatin* was down-regulated by 3.75-, 3.22-, 1.023-, and 2.24-fold in SDBH2.5, SDBH5, SDBH7.5, and SDBH10 groups, respectively. 

### 3.6. Histopathological Findings and Morphometric Measures

**Figure 4:**
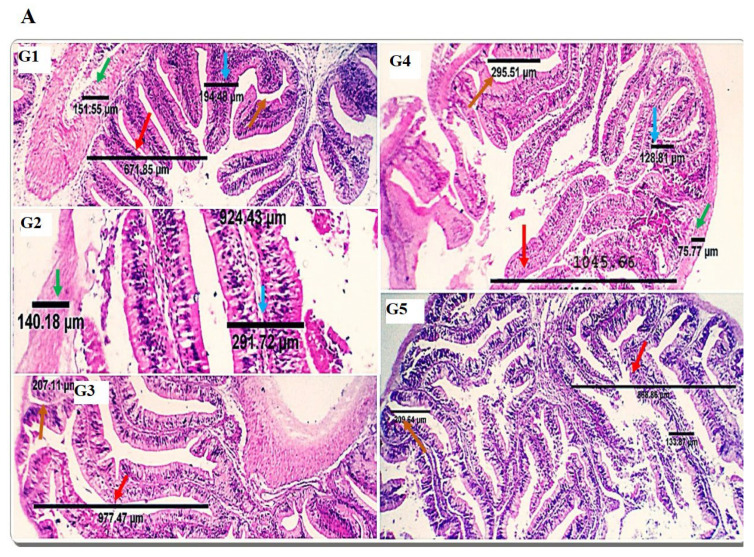

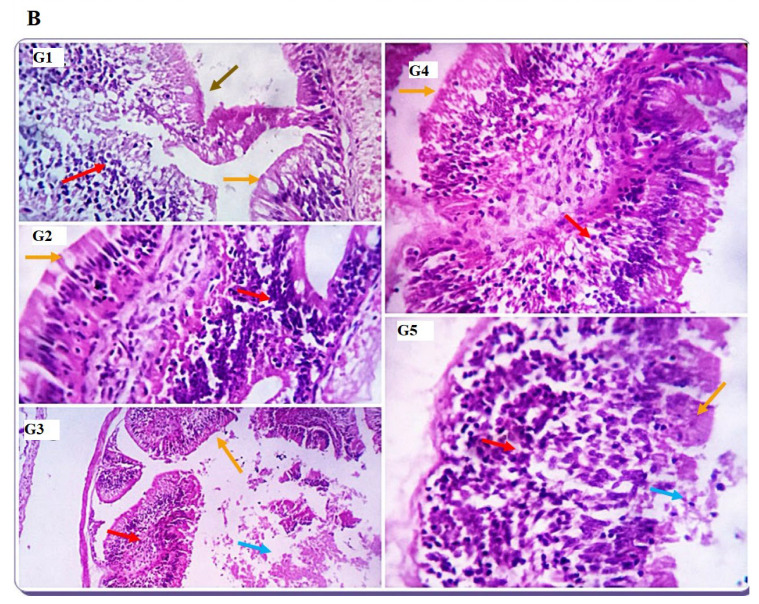
Photomicrographs demonstrating the histomorphological changes in the anterior (**A**) and posterior (**B**) intestines of the control (1) and treatment groups (2–5) with variable dimensional changes regarding villous length (red arrows), villous width (blue arrows), the crypt of Lieberkühn length (orange arrows), muscular coat thickness (green arrows), and goblet cell populations (black star). Mucosal folds of the posterior intestine (brown arrows) and lymphocytic populations (red arrows) are seen. G1: SDBH0, G2: SDBH2.5, G3: SDBH5, G4: SDBH7.5, G5: SDBH10. H&E. scale bar 50 and 100 µm.

The normal histological structure was shown in the examined anterior intestine of the SDBH0 group. Examined sections from the intestine of treated groups (SDBH2.5-10) showed comparative variability among groups regarding the villous length, villous width, crypts of Lieberkühn length, muscle coat thickness, and the number of villous and crypt goblet cells (Figure 4A).

Examined sections from the posterior intestine of the SDBH0 group revealed a typical histological structure. The posterior intestine of the treated groups was similar to the control group. The lamina epithelialis showed mild to moderate lymphocyte infiltration of normal active histologic morphology and population in the SDBH7.5 group. Still, the number was more countable and beyond the average level in the SDBH10 group (Figure 4B).

Table 5 presents the morphometric measures of the anterior intestine of fish fed the treated diets. The villous height (VH), villous width (VW), villous height: crypt depth ratio (VH: CD), and muscular coat thickness (MCT) were increased linearly and quadratically in all SDBH inclusion levels compared to SDBH0 (*p* < 0.01); the SDBH7.5 group exhibited the best morphometric characteristics. The crypt depth (CD) was decreased linearly and quadratically in the SDBH 2.5–7.5 groups and higher in the SDBH10 group compared to the SDBH0 group (*p* < 0.01). The highest goblet cell count was seen in the SDBH7.5 group (7–8/high power field (HPF)), and the lowest counts were seen in the SDBH2.5 and SDBH10 groups (4–5, 3–4/HPF, respectively). The control group (SDBH0) showed 5–6 cells/HPF (Figure 4A).

**Figure 5:**
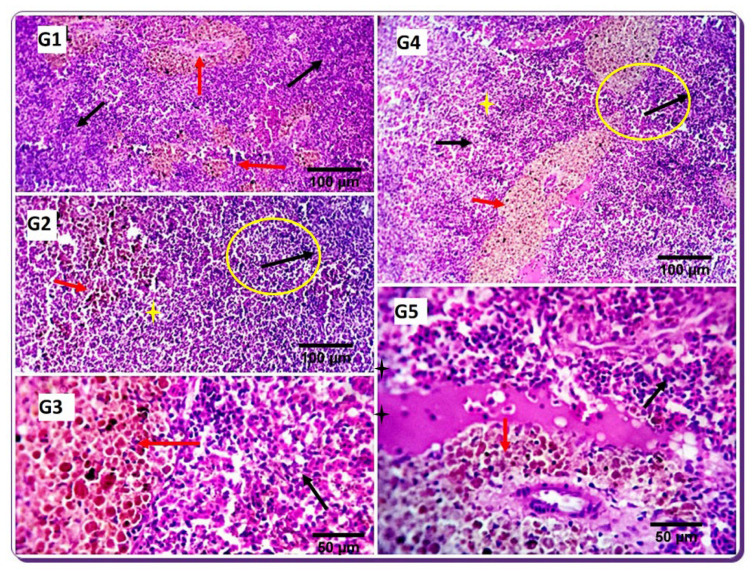
Photomicrographs demonstrating the histomorphological structures in the spleen of control fish (1) and the spleen of treatment groups (2–5). The spleen of the control fish shows mild proliferative clusters of melanomacrophages (red arrows), and the white pulp comprising mainly lymphoid cells usually encircles small arterioles, occasionally assuming a nodular pattern (black arrows). The spleen of the treatment groups shows histomorphological structures similar to the control group. However, the lymphoid components formed clusters or different nodular arrangements mainly near the small blood vessels (yellow circles and black arrows), and the splenic cords (yellow stars) are outstanding, especially in groups 2 and 4 (black arrows). Moderately activated MM centers were observed in groups 3 and 4 (red arrows). Mild interstitial edema (black star) and mild to moderate melanomacrophage center activations (red arrow) were visible in G5. G1: SDBH0, G2: SDBH2.5, G3: SDBH5, G4: SDBH7.5, G5: SDBH10. H&E. scale bar 50 and 100 µm.

In the current study, spleens of the SDBH0 group exhibited mild proliferative clusters of melanomacrophages (MM), a specialized immune cell type prevalent in the spleen, both perivascular and interstitial. The spleen of the treatment groups showed histomorphological structures like the SDBH0 group. The lymphoid components formed clusters or ill-defined nodular arrangements mainly near the small blood vessels, particularly in the SDBH2.5 and SDBH7.5 groups. Moderate activation of the MM centers was observed in the SDBH5 and SDBH7.5 groups. Moreover, mild interstitial edema and mild to moderate melanomacrophage center activations were noted in the SDBH10 group (Figure 5).

### 3.7. Immunological Parameters

The lysozyme activity was raised by dietary inclusion of SDBH, with the highest values recorded in the SDBH5 group (*p* < 0.05). The nitric oxide level was linearly and quadratically increased in the SDBH5 group (*p* < 0.05). The complement 3 level was raised in the SDBH5 group (linear, *p* = 0.01). The phagocytic % and the phagocytic index were linearly increased by increasing the SDBH inclusion level (*p* < 0.01) (Table 6).

### 3.8. Immune-Related Gene Expression in the Spleen

**Figure 6:**
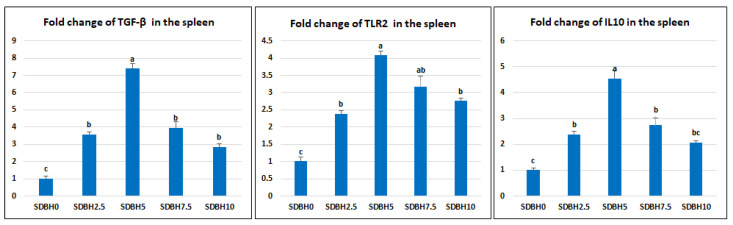
Impact of dietary SDBH inclusion on the immune-related genes (transforming growth factor-beta (*TGF-β*), Toll-like receptor 2 (*TLR2*), and interleukin 10 (*IL-10*)) in the spleen of *O. niloticus.* ^a,b,c^ bars with different lowercase letters are significantly different (*p* < 0.05).

*TGF-β, TLR2,* and *IL10* were up-regulated by SDBH inclusion, with the highest expression observed in the SDBH5 group (linear and quadratic *p* < 0.01). Compared to the control group (SDBH0), the immune-related genes were boosted by 3.57-, 7.39-, 3.96-, and 2.83-fold for *TGF-β,* 2.38-, 4.08-, 3.17-, and 2.76-fold for *TLR2,* and 2.38-, 4.54-, 2.73-, and 2.06-fold for *IL10* in SDBH2.5, SDBH5, SDBH7.5, and SDBH10, respectively (Figure 6).

### 3.9. Economic Efficiency

The total feed intake per tank was not significantly differed between groups (*p* > 0.05). The feed costs and feed costs/kg gain were linearly decreased in the SDBH7.5 and SDBH10 diets (*p* < 0.01) (Table 7).

## 4. Discussion

The present study assessed the possible use of spray-dried bovine hemoglobin as a novel protein source in Nile tilapia diets. The study evaluated the effects of SDBH inclusion on growth, growth-related genes, digestive enzyme activity, blood biochemical parameters, intestinal histomorphology, immune status, and immune-related genes in Nile tilapia, *O. niloticus.* The results of the present study reported increased FBW, TWG, SGR, and PER by increasing the level of SDBH inclusion. Dietary SDBH7.5 and SDBH10 decreased daily feed intake relative to body weight and FCR. The SDBH10 group showed the highest performance compared to other groups. Yao et al. [26] showed that the optimum dietary inclusion with hemoglobin powder protein was 17.3% in hybrid grouper (*Epinephelus fuscoguttatus × Epinephelus lanceolatus*) based on the maximum response weight gain. They also reported that the highest inclusion levels (43.38 and 52.05%) showed a reduction in the growth performance, feed intake, feed utilization, and feeding-related gene expression of the fish due to insufficient essential amino acids (threonine and arginine) and poor palatability and gut growth. Ding et al. [27] showed a reduced growth rate of largemouth bass (*Micropterus salmoides*) with dietary inclusion of chicken hemoglobin powder up to 7.66%, which may be due to the effect of chicken hemoglobin powder inclusion on the feed intake and the protein and amino acids’ digestibility. Chookird et al. [28] reported decreased shrimp growth with increasing the level of hemoglobin powder dietary inclusion (4.26–34.17%). De Araújo et al. [29] demonstrated the effects of dietary spray-dried plasma (0, 16.6, 33.2, 49.7, and 66.3 g kg−1) on the growth and intestinal health of Nile tilapia. They reported improved fish growth, hematological profile, and intestinal health, and the optimal level of inclusion was 51.83 g kg^−1^. Gisbert et al. [10] indicated that dietary spray-dried plasma protein from porcine (3 and 6%) increased the growth of gilthead sea bream (*Sparus aurata*) fingerlings, where the body weight was increased by 10.5% in fish fed 3% spray-dried plasma protein compared to the control group. Twahirwa et al. [30] reported decreased intestinal trypsin activity and lower amylase in black carp, *Mylopharyngodon piceus*, with increasing dietary blood meal levels (40 and 100%).

The somatic growth, feed consumption, and energy-storing in vertebrates are highly maintained evolutionary modifications controlled by peripheral signals, such as *IGF-1*, leptin, and several hypothalamic neuropeptides, which regulate the appetite [31,32]. The growth hormone (GH) and IGF-1 axis regulate the most significant peripheral endocrine factors related to growth and nutritional status in fish [33,34]. Pituitary GH promotes the synthesis and release of IGF-1 from the liver, which affects skeletal muscle and the gonads to motivate somatic growth [35,36]. Hence, the GH–IGF-1 system is crucial for growth regulation, and thus IGF-1 concentration increases or decreases in anabolic or catabolic conditions, respectively [34,37]. 

Myostatin (MSTN), or growth and differentiation factor 8 (GDF-8), a member of the Transforming Growth Factor-β (TGF-β) superfamily, was first detected in mice by McPherron et al. [38] and controlled the growth of skeletal muscle in various mammalian species adversely. MSTN plays an inhibitory role in developing and managing skeletal muscle size. Consequently, mutations that deactivate or decrease the protein activity markedly increase muscle size [38,39]. Myostatin inhibits myoblast differentiation and proliferation [40,41], which was achieved by downregulation of the myogenic gene expression [40,42]. The MSTN gene has been identified in various vertebrate species, including numerous fish species [43,44,45].

Consequently, the increased fish growth in SDBH-included groups in the current study may be attributed to the increased growth hormone level and decreased leptin hormone level that was noted by increasing the SDBH inclusion level, in addition to increasing the digestive enzymes’ activity (amylase and protease), which results in increased nutrient digestibility. Leptin is produced in the white adipose tissue and regulates energy homeostasis, spending, feed intake, reproduction, and obesity [46,47]. In fish, leptin is produced and distributed in the liver, with the highest leptin expression noted by [48,49,50,51]. It has been shown that leptin secretion in fish may be altered due to diet composition [52]. In the study of Liang et al. [53], grass carp (*Ctenopharyngodon idellus)* fed a plant-protein-based diet showed reduced leptin levels compared to a fishmeal-based diet. Moreover, leptin synthesis is regulated by pituitary growth hormone; increasing the level of GH leads to reduced leptin secretion in fish [54]. In the current study, although leptin secretion decreased with increasing the level of SDBH inclusion, the daily feed intake relative to the BW was decreased in the SDBH7.5 and SDBH10 groups. We can explain this by the effect of dietary SDBH in improving the feed conversion rate, nutrient utilization, and uptake rather than increasing the feed intake. This has the advantage of fish eating less feed and converting it to more weight gain.

The increased growth in SDBH-included groups also may be due to the up-regulation of *IGF-1* and down-regulation of *myostatin*. The results showed that the expression of IGF-1 was upregulated in the SDBH-included groups in a linear trend up to the inclusion level of 7.5%, then down-regulated but still higher than the non-included group. The IGF-1 pathway can regulate skeletal muscle protein synthesis via rapamycin (TOR) activation and inhibit skeletal muscle protein breakdown through the phosphorylation of Forkhead box O (FoxO) transcription factors [55,56]. So the highest inclusion levels (10%) did not stimulate more expression of IGF-1 in the muscle. This influence is correlated with the level of protein absorption in the intestine that AA and peptide transporters regulate. The current study found that the expression of these transporters was increased up to the inclusion level of 7.5% and then reduced at the highest level (10%). So the level of 10% SDBH in the diets might result in extra protein in the intestine during digestion without absorption. This indicated that IGF-1 expression might be modified by muscle AA [57].

The serum glucose level was decreased in the SDBH7.5 and SDBH10 groups. The hypoglycemic effect of SDBH is due to the reduced daily feed intake relative to BW in these groups and the up-regulation of mRNA expression of *insulin growth factor-1* (*IGF-1*); its hypoglycemic effect is reported in fish [58]. 

During the digestive process, dietary proteins are broken down to free amino acids and di- or tripeptides in the intestinal lumen; then, they are absorbed through AA and peptide transporters, such as peptide transporter 1 (PepT1, SLC15A1), proton-coupled oligopeptide transporter (SLC15A2, peptide transporter 2, PepT2), and SLC26A6 (PAT-1, putative anion transporter-1) [59,60]. Peptide transporter 1 (PepT1) could transport all potential 400 dipeptides and 8000 tripeptides [61,62]. The entire intestine may be implicated in peptide absorption because PepT1 has been well-expressed in the fish intestine from proximal to distal parts [63,64,65]. The present results showed up-regulation in the intestinal mRNA expression of SLC15A2 and SLC26A6 in SDBH-included groups. The highest expression was reported in the SDBH7.5, then began to downregulate in the highest inclusion level (10%) but was still higher than the non-included group. This means that the highest protein absorption was in the inclusion levels of 2.5–7.5% SDBH, then decreased at the highest level (10%). Inconsistent with our results, the mRNA expression of PepT1 in the proximal and distal intestine was down-regulated in high levels of fish protein hydrolysate in turbot and yellow catfish (*Pelteobagrus fulvidraco*) [66,67,68]. Because PepT1 can be controlled by its transmissible substrates [60], the present results indicated that the highest level of SDBH in the diets (10%) might result in an extra of di- or tripeptides in the intestine during digestion. 

The gut is the primary feed digestion and nutrient uptake site, and optimal nutrient utilization relies on its functional efficacy [69]. Because dietary protein affects the structure of intestinal microvilli [70], research studies have investigated the effects of different dietary protein sources on the histomorphology of the gut. In the current study, dietary inclusion with SDBH showed a significant increase in the intestinal VH, VW, VH: CD, and MCT, with the highest values in the SDBH7.5 group and then decreased in the SDBH10 group. These results confirm the results of the intestinal mRNA expression of SLC15A2 and SLC26A6. This implies that the gut of Nile tilapia may badly affect the uptake and absorption of free AA and peptides from the high level of SDBH through the regulation of AA and peptide transporters. It has been reported that high inclusion levels of hemoglobin powder protein (43.38 and 52.05%) in hybrid grouper decreased the enterocyte and microvillus height of the foregut and midgut and may damage gut health [26]. Amer et al. [71] reported improved gut development by dietary inclusion with whey protein concentrate of up to 5% in Nile tilapia diets. Gisbert et al. [10] reported no significant effect of spray-dried plasma protein (SDPP) on the intestinal villi width and length, with an increased number of goblet cells in the gilthead sea bream fed SDPP by a level of 6%, followed by 3%.

The first line of defense against infection is innate immunity, considered primitive and, thus, the general form of host defense [72]. Interleukin (IL-10) is an immune-regulating cytokine that has many functions. It was primarily recognized as a cytokine synthesis inhibitory factor [73]. *IL-10* is considered an anti-inflammatory cytokine and shows a vital function in controlling inflammation [74]. Transforming growth factor-beta (*TGF-β*) is a pleiotropic cytokine released during an immune response with significant immunomodulatory functions in the regulation, differentiation, proliferation, migration, survival, activation, and deactivation of macrophages and other immune cells [75]. TGF-β is mainly produced by macrophages, platelets, and fibroblasts [76]. *Toll-like receptors* (*TLRs*) are a significant component of innate immunity. TLR2 is concerned with recognizing specific microbial structures such as zymosan, lipoteichoic acid (LTA), and peptidoglycan (PGN). Its binding leads to primary myeloid differentiation response gene 88 (MyD88), a dependent signaling pathway to induction of numerous cytokines [77]. The current study showed increased lysozyme activity, NO, and C3 serum levels by SDBH inclusion, with the highest values recorded in the SDBH5 group. The phagocytic % and the phagocytic index were increased by increasing the SDBH inclusion level. TGF-β, TLR2, and IL-10 were up-regulated in all SDBH-included groups compared to the SDBH0 group, with the highest expression observed in the SDBH5 group. These results demonstrated the immunomodulating effect of SDBH in Nile tilapia. The improved immune responses may be attributed to the high immunoglobulin content in SDBH [78]. Gisbert et al. [10] indicated higher nonspecific serum immune responses in gilthead sea bream fingerlings fed spray-dried plasma protein than the control group. Twahirwa et al. [30] detected reduced lysozyme activity in black carp (*Mylopharyngodon piceus*) fed bloodmeal-included diets (20–100%) compared with the control group, while the complement C3 and C4 was not different from the control group.

Concerning the economic efficiency of the diets, the feed costs and feed cost/kg gain were decreased in the highest SDBH inclusion levels (SDBH7.5 and SDBH10 diets), which was a consequence of the improved growth performance and the lower price of SDBH in comparison with fishmeal.

## 5. Conclusions

From the obtained results, we concluded that SDBH could be efficiently used as a protein source in diets of Nile tilapia up to 10%. Dietary SDBH increases fish growth by improving gut histoarchitecture, increasing the serum growth hormone level, decreasing the serum leptin hormone level, and increasing the activity of the digestive enzymes (amylase and protease), up-regulation of *IGF*-*1* and protein and amino acid transporters, and down-regulating the expression of *myostatin*. Moreover, SDBH enhances the immune status of fish through increasing lymphoid elements in the spleen tissues, increasing the levels of immune indices (lysozyme activity, NO, C3, phagocytic %, and the phagocytic index), up-regulation of gene expression of *TGF-β*, *TLRs*, and *IL10*. The economic efficiency of the diets (feed costs and feed cost/kg gain) was improved by increasing the SDBH inclusion level.

## Figures and Tables

**Table 1:** Proximate chemical composition of the experimental diets (% on a dry weight basis).

Ingredients	SDBH0	SDBH2.5	SDBH5	SDBH7.5	SDBH10
Yellow corn ^1^	22	22	22.5	23.45	24.4
Fish meal ^2^, 70.7% CP	20	17.5	15	12.5	10
SDBH ^3^	0	2.5	5	7.5	10
Soybean meal ^4^, 49% CP	26	26	24.45	23	21.5
Wheat flour	10	10	10	10	10
Corn gluten ^5^, 67% CP	7	7	7	7	7
Fish oil	7	7	7.5	7.5	7.5
Wheat bran	5	5	5	5	5
Dicalcium phosphate	0	0	0.5	1	1.5
Methionine	0	0	0.05	0.05	0.1
Vitamins and minerals mixture	3	3	3	3	3
Proximate composition (%)					
Crude Protein	35.86	36.39	36.23	36.13	36.06
Fat	10.92	10.68	10.93	10.71	10.48
NFE ^6^	43.13	43.2	43.67	44.53	45.39
Crude fiber	3.47	3.46	3.36	3.28	3.19
Ash	6.6	6.24	5.78	5.34	4.89
Lysine	2.07	2.17	2.22	2.28	2.34
Methionine	0.75	0.72	0.73	0.69	0.7
GE MJ/kg ^7^	20.85	20.90	21.02	21.04	21.06

^1^ Ukrainian yellow corn, Flexs Trading LLC, Odessa, Ukraine. ^2^ Argentinean Fish Meal by Coomarpes Ltd. Mar del Plata, Argentina. ^3^ Spray-dried bovine hemoglobin powder, ACTIPRO^®^ 95 BHS, Zwevezele, Belgium. Chemical composition of SDBH (% on dry matter basis): Crude protein 92%, ash 4%, fat 0.4%, Crude fiber 0.5%, Calcium 0.02%, Phosphorus 0.3%, Nitrogen free extract 3.1%. ^4^ US Soybean Meal, AG Food Commodities, Washington, DC, USA. ^5^ US Corn Gluten, Global Elaion Trading LLC, Belcamp, MD, USA. ^6^ Nitrogen free extract, determined by difference = 100 − (protein %+ fat %+ crude fiber %+ ash %). ^7^ Gross energy (GE) was estimated as 17.0 KJ/g NFE, 39.5 KJ/g lipids, and 23.6 KJ/g protein [5]. CP: crude protein.

**Table 2:** Primers of growth and immune-related genes for real-time quantitative PCR amplification.

Primer	Forward Primer	Reverse Primer	Accession Number
*β-Actin*	CCACCCAAAGTTCAGCCATG	ACGATGGAGGGGAAGACAG	XM_003443127.5
*TGF-β*	CCAGAGCAGAGCTACGGATG	CCAGGTCTGCAGAGGTTCAG	NM_001311325.1
*TLR2*	TGGCACAGGACACTTAAGCA	GCGACGAGCACTGAGATACT	XM_019360109.2
*IL-10*	CATCAGCATTTCTGTGGACCAG	TTCTTGAGCCTGACGGGGAA	KP645180.1
*SLC15A2*	CTGCGAACGCTTCTCCTACT	CGCTGAAAGCATGGTAGACA	XM_005475385.4
*SLC26A6*	GAGAGAGAGGAGCTCAGGGAG	TCTCTGCCATGGCTATTGGG	XM_005478355.3
*Myostatin*	GCCATCCAGCACCCTACTTT	GGCTGTTTGTGGCCTCTACT	XM_003458832.5
*IGF-1*	TCAAGAGTGCGATGTGCTGT	AAACTGCAGCGTGTCTACCA	NM_001279503.1

β-actin: beta-actin, TGF-β: transforming growth factor-beta, TLR2: Toll-like receptor 2, IL-10: interleukin 10, SLC15A2: solute carrier family 15 members 2, SLC26A6: solute carrier family 26 members 6, IGF-1: Insulin-like Growth Factor-1.

**Table 3:** Impact of dietary SDBH inclusion on the growth performance of *O. niloticus.*

	SDBH0	SDBH2.5	SDBH5	SDBH7.5	SDBH10	SEM	Linear Reg.	Quadratic Reg.
IBW (g/fish)	32.59	32.38	32.36	32.26	32.28	0.05	0.12	0.44
FBW (g/fish)	83.36 ^b^	86.25 ^b^	88.18 ^ab^	89.31 ^ab^	94.00 ^a^	1.24	<0.01	0.58
ADWG (g/fish/day)	0.73 ^b^	0.77 ^b^	0.80 ^ab^	0.81 ^ab^	0.88 ^a^	0.01	<0.01	0.64
TWG (g/fish)	50.76 ^b^	53.87 ^b^	55.83 ^ab^	57.05 ^ab^	61.72 ^a^	1.28	<0.01	0.64
ADFI (g/g BW/day)	0.76	0.74	0.76	0.67	0.66	0.01	0.02	0.33
FCR	1.25 ^a^	1.18 ^ab^	1.21 ^ab^	1.05 ^ab^	1.01 ^b^	0.03	<0.01	0.38
PER	2.49 ^b^	2.59 ^ab^	2.53 ^ab^	2.92 ^ab^	3.06 ^a^	0.08	<0.01	0.23
SGR	1.34 ^b^	1.40 ^ab^	1.43 ^ab^	1.45 ^ab^	1.53 ^a^	0.02	<0.01	0.83

^a,b^ Means with different superscripts in the same row were significantly different (*p* < 0.05) as the main effect. The regression was considered significant when *p* < 0.05. IBW, initial body weight; FBW, final body weight; ADWG, average daily weight gain; TWG, total body weight gain; ADFI, average daily feed intake; FCR, feed conversion ratio; PER, protein efficiency ratio; SGR, specific growth rate.

**Table 4:** Impact of dietary SDBH inclusion on the blood biochemical parameters of *O. niloticus.*

	SDBH0	SDBH2.5	SDBH5	SDBH7.5	SDBH10	SEM	Linear Reg.	Quadratic Reg.
GH (pg/dl)	17.93 ^b^	20.25 ^b^	23.38 ^b^	45.66 ^a^	51.97 ^a^	4.75	<0.01	0.01
Leptin (ng/mL)	6.95 ^a^	5.04 ^b^	4.04 ^bc^	2.99 ^cd^	2.48 ^d^	0.53	<0.01	0.01
Glucose (mg/dl)	150.90 ^a^	158.45 ^a^	140.05 ^a^	108.90 ^b^	102.85 ^b^	7.54	<0.01	0.02

^a,b,c,d^ Means with different superscripts in the same row were significantly different (*p* < 0.05) as the main effect. The regression was considered significant when *p* < 0.05. GH: growth hormone.

**Table 5:** Impact of dietary SDBH inclusion on the intestinal histomorphometric measures (um) of *O. niloticus.*

	SDBH0	SDBH2.5	SDBH5	SDBH7.5	SDBH10	SEM	Linear Reg.	Quadratic Reg.
VH	671.33 ^e^	823.07 ^d^	966.70 ^b^	1017.90 ^a^	862.18 ^c^	27.85	<0.01	<0.01
VW	126.67 ^d^	131.87 ^d^	192.50 ^b^	277.83 ^a^	155.87 ^c^	12.79	<0.01	<0.01
CD	292.57 ^b^	204.63 ^c^	208.63 ^c^	173.60 ^d^	321.64 ^a^	13.02	<0.01	<0.01
VH: CD	2.29 ^e^	4.02 ^c^	4.63 ^b^	5.86 ^a^	2.68 ^d^	0.3	<0.01	<0.01
MCT	51.67 ^e^	76.83 ^d^	133.40 ^b^	149.87 ^a^	116.60 ^c^	8.34	<0.01	<0.01

^a,b,c,d,e^ Means with different superscripts in the same row were significantly different (*p* < 0.05) as the main effect. The regression was considered significant when *p* < 0.05. VH: villous height, VW: villous width, MCT: muscular coat thickness. VH: CD, villous height: crypt depth ratio.

**Table 6:** Impact of dietary SDBH inclusion on the immune status of *O. niloticus.*

	SDBH0	SDBH2.5	SDBH5	SDBH7.5	SDBH10	SEM	Linear Reg.	Quadratic Reg.
Lysozymes (ng/mL)	0.12 ^c^	0.21 ^bc^	0.52 ^a^	0.41 ^ab^	0.42 ^ab^	0.05	<0.01	0.02
C3 (mg/dl)	30.17 ^ab^	23.49 ^ab^	34.02 ^a^	18.86 ^ab^	9.92 ^b^	3.12	0.01	0.09
NO (mg/mL)	2.29 ^b^	4.41 ^ab^	11.69 ^a^	9.81 ^ab^	6.64 ^ab^	1.23	0.02	0.01
Phagocytic %	52.50 ^c^	60.00 ^bc^	67.50 ^ab^	72.50 ^ab^	78.00 ^a^	3.12	<0.01	0.52
Phagocytic index	1.82 ^b^	1.93 ^b^	2.34 ^ab^	2.75 ^ab^	3.26 ^a^	0.18	<0.01	0.27

^a,b,c^ Means with different superscripts in the same row were significantly different (*p* < 0.05) as the main effect. The regression was considered significant when *p* < 0.05. NO: Nitric oxide, C3: complement 3.

**Table 7:** Impact of dietary SDBH inclusion on the economic efficiency of the diets.

	SDBH0	SDBH2.5	SDBH5	SDBH7.5	SDBH10	SEM	Linear Reg.	Quadratic Reg.
TFI (kg/tank)	0.95	0.96	1.01	0.9	0.93	0.01	0.40	0.41
Feed costs (USD)	0.80	0.78	0.80	0.69	0.69	0.02	0.02	0.31
Feed costs/kg gain	1.02 ^a^	0.95 ^ab^	0.94 ^ab^	0.79 ^bc^	0.73 ^c^	0.03	<0.01	0.41

^a,b,c^ Means with different superscripts in the same row were significantly different (*p* < 0.05) as the main effect. The regression was considered significant when *p* < 0.05. The cost of one kg of fish meal was 1.66 USD, and the cost of one kg of SDBH was 0.75 USD. The cost of one kg of diet was 0.83, 0.81, 0.79, 0.76, and 0.74 USD for SDBH0, SDBH2.5, SDBH5, SDBH7.5, and SDBH10 diets, respectively.

## Data Availability

Data contained in the article.
